# Episodic fevers and vasodilatory shock mimicking urosepsis in a patient with HIV-associated multicentric Castleman’s Disease: a case report

**DOI:** 10.1186/s12879-016-1378-5

**Published:** 2016-02-01

**Authors:** Stephanie Anderson, Sarah C. Sasson, Frederick J. Lee, Wendy Cooper, Stephen Larsen, Roger Garsia

**Affiliations:** 1 Clinical Immunology Department, Royal Prince Alfred Hospital, Sydney, NSW Australia; 2 Sydney Medical School, University of Sydney, Sydney, NSW Australia; 3 Haematology Department Royal Prince Alfred Hospital, Sydney, NSW Australia; 4 Tissue Pathology and Diagnostic Oncology, Royal Prince Alfred Hospital, Sydney, NSW Australia; 5 School of Medicine, University of Western Sydney, Parramatta, NSW Australia; 6 Level 6 Laboratory Services Building, Royal Prince Alfred Hospital, Missenden Rd Camperdown, Sydney, NSW Australia

**Keywords:** Multicentric Castleman’s disease, MCD, HHV-8, Human herpes virus-8, HIV, Fevers, Shock, Rituximab, Systemic inflammatory response syndrome, SIRS

## Abstract

**Background:**

Multicentric Castleman’s disease (MCD) is a pre-malignancy that presents with lymphadenopathy and features of systemic inflammation. Human immunodeficiency virus (HIV)-associated MCD is associated with human herpesvirus-8 (HHV-8) infection. If untreated MCD has a relapsing and remitting course that is eventually fatal.

**Case presentation:**

A 67-year-old man had six hospital admissions over 20 months characterised by fever, urinary frequency and CRP >100 mg/L. The final admission was complicated by hypotension requiring intensive care unit admission and ionotropic support. His history included HIV and Hepatitis B virus (HBV) co-infection on suppressive therapy. Each presentation was managed as presumed urosepsis with use of empirical antibiotics, however numerous blood and urine cultures failed to identify a pathogen. A bone-marrow aspirate and trephine found no evidence of haematological malignancy. A positron emission tomography scan found active lymph nodes, one of which was biopsied and found to contain the plasma-cell variant of Castleman’s disease. Ultimately the cause for the recurrent presentations was attributed to progressive MCD. The patient received rituximab monotherapy and has had no further related admissions.

**Conclusions:**

MCD should be considered in patients with chronic HIV infection presenting with recurrent sepsis-like episodes and/or vasodilatory shock, particularly if no pathogen is identified or lymphadenopathy is evident.

## Background

Castleman’s disease is a rare, pre-malignant, polyclonal lymphoproliferative disorder of unknown aetiology [1]. Estimates suggest a prevalence of 2.4 per million [2] with a male and Caucasian preponderance and a median age of 55 years [2]. Unicentric Castleman’s disease typically involves a single lymph node that may be cured by excision. In contrast, multicentric Castleman’s disease (MCD) is an aggressive condition marked by generalised lymphadenopathy, extra-nodal disease, and clinical features of a systemic inflammatory response [2].

Early in the human immunodeficiency virus (HIV) pandemic clinicians noted an association between Kaposi’s sarcoma and MCD. It was subsequently recognised that both conditions shared an association with human herpes virus-8 (HHV-8) [3]. A recent study of MCD cases from two centres found 15 % were HIV-associated and 17 % HHV-8-associated, with nearly all cases of HIV-associated MCD expressing histologically detectable HHV-8 antigen [4]. The proposed pathogenesis of HIV-associated MCD is that immunodeficiency promotes viral replication of HHV-8 in lymph node plasmablasts, leading to cellular transformation.

The majority of HIV-associated MCD are plasma cell-variant or mixed plasma-cell-hyaline variant [3]. Coexistence of Kaposi’s sarcoma has been found in 40–75 % of cases [3, 5, 6] and an association between MCD and non-Hodgkin’s lymphoma and primary effusion lymphoma has been recognised [3]. In the absence of treatment, MCD has a relapsing and remitting course that is eventually fatal [4, 7].

MCD commonly presents with a syndrome of fevers, fatigue, night sweats, weight loss, lymphadenopathy and hepatosplenomegaly. Laboratory abnormalities include raised serum C-reactive protein (CRP) and interleukin (IL)-6, cytopenias and polyclonal hypergammaglobulineamia. Computed tomography (CT) imaging typically shows diffuse adenopathy and splenomegaly. The diagnosis is confirmed by histological examination of lymph node tissue.

The pathogenesis of MCD remains poorly understood, however infection of immunoblasts with HHV-8 appears to trigger an intense immune response and marked production of host-derived IL-6. HHV-8 also encodes an early homologue of IL-6 (vIL-6) [8] and this plays a pathogenic role by stimulating B-cell proliferation and oncogenesis [9]. Elevated circulating IL-6 derived from both host and HHV-8 is thought to contribute to the manifestations of a systemic inflammatory response [10, 11] and related laboratory findings [3]. Kaposi sarcoma herpesvirus-inflammatory cytokine syndrome (KICS) has recently been described which can occur in the setting of HHV-8 and elevated IL-6 in the absence of the histological finding of MCD [4, 12, 13].

Here we report a case that aims to illustrate the challenges surrounding the diagnosis of MCD due to non-specific symptoms that relapse and remit and may resemble other conditions, and a lack of standardised diagnostic criteria. Additionally, current treatment strategies and rationale are summarised. The patient had a background of chronic, well-controlled HIV-infection and had multiple hospital presentations characterised by fever and urinary frequency that mimicked urosepsis. No pathogenic organisms were isolated from multiple urine and blood cultures. The last episode was characterised by systemic inflammatory response syndrome (SIRS) [14] and vasodilatory shock, requiring intensive care unit (ICU) admission and ionotropic support. Following diagnosis and treatment for his MCD his fevers abated promptly with no further episodes of fevers or shock.

## Case presentation

A 67 year old man of Mediterranean descent presented with vomiting and diarrhoea. His past medical history included advanced HIV-1 infection diagnosed 4 years prior and hepatitis B virus (HBV) co-infection on combination antiretroviral therapy (ART) targeting both viruses, gastro-oesophageal reflux disease (GORD), vitamin D deficiency and depression. Medications at presentation were: atazanavir 400 mg, zidovudine 250 mg, etravirine 200 mg BD, raltegravir 400 mg BD, ranitidine 300 mg, fluconazole 200 mg, sertraline 50 mg and cholecalciferol 25 μg.

On examination, he had a low grade fever to 37.7 °C and a normal cardiopulmonary examination. He had palpable discrete cervical and axillary lymphadenopathy. There was generalised abdominal tenderness and hepatosplenomegaly without ascites.

**Fig. 1:**
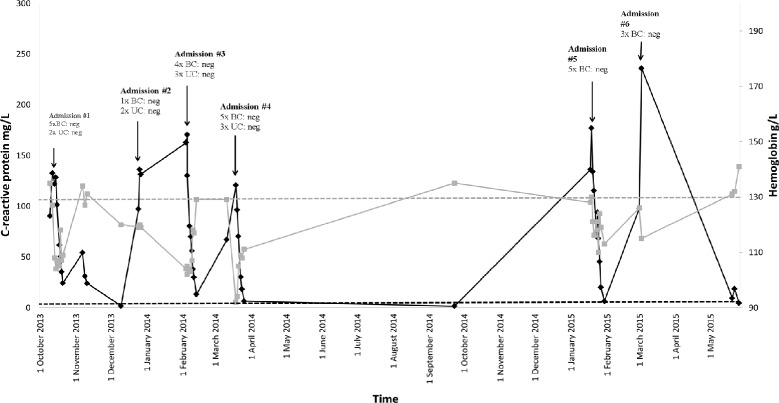
Summary of hospital admissions spanning 20 months. Data from six hospital admissions between October 2013 and May 2015 are shown. C-reactive protein is shown on the left y-axis (*black*) with upper limit of normal 5 mg/L (*black dashed line*). Haemoglobin is shown on the right y-axis (*gray*) with lower limit of normal 130 g/L (*gray dashed line*). BC blood culture; UC urine culture

Laboratory investigations on initial presentation are shown in Table 1. The most salient finding was an elevated CRP of 132 mg/L (Fig. 1, Admission #1). The CD4 T-cell count was 0.34 x 10^9^ cells/L and the HIV-1 RNA plasma viral load was undetectable to a level of 37 copies/mL. Five blood cultures and two urine cultures returned no growth. Stool microscopy, culture and serum cytomegalovirus (CMV) DNA quantitation were negative.

**Table 1:** Laboratory values for admission #1 and admission #4 are shown with abnormal results in bold. ND not done

	Admission #1 September 2013	Admission #4 March 2015	Reference range
White cell count	**3.6 × 10** ^**9**^ **/L**	5.3 **×** 10^9^/L	4.0–10.0 x10^9^/L
Haemoglobin	134 g/L	**126 g/L**	130–170 g/L
Platelets	**135 × 10** ^**9**^ **/L**	**91 × 10** ^**9**^ **/L**	150–400 x10^9^/L
Packed cell volume	0.40 L/L	**0.37 L/L**	0.40–0.50 L/L
Mean corpuscular volume	**107.0 fL**	**111.0 fL**	80–100 fL
Red cell count	**3.77 × 10** ^**12**^ **/L**	**3.37 × 10** ^**12**^ **/L**	4.50–5.50 x10^12^.L
Mean corpuscular haemoglobin	**35.5 pg**	**37.4 pg**	27.0–32.0 pg
Mean corpuscular haemoglobin concentration	332 g/L	338 g/L	315–355 g/L
Red cell distribution width	13.5 %	14.7 %	11.6–14.0 %
Neutrophils	2.7 × 10^9^/L	3.2 × 10^9^/L	2.0–7.0 x10^9^/L
Lymphocytes	**0.5 × 10** ^**9**^ **/L**	1.0 × 10^9^/L	1.0–3.0 x10^9^/L
Monocytes	0.2 × 10^9^/L	0.9 × 10^9^/L	0.2–1.0 x10^9^/L
Eosinophils	0.1 × 10^9^/L	0.1 × 10^9^/L	0.0–0.5 x10^9^/L
Basophils	0.0 × 10^9^/L	0.0 × 10^9^/L	0.0–0.1 x10^9^/L
Sodium	137 mmol/L	135 mmol/L	135–145 mmol/L
Potassium	4.0 mmol/L	4.6 mmol/L	3.5–5.0 mmol/L
Chloride	99 mmol/L	97 mmol/L	97–109 mmol/L
Bicarbonate	**22 mmol/L**	**23 mmol/L**	24–32 mmol/L
Urea	7.6 mmol/L	**8.4 mmol/L**	3.0–8.0 mmol/L
Creatinine	98 μmol/L	103 μmol/L	70–110 μmol/L
Estimated glomerular filtration rate	69 mL/min/1.73 m^2^	64 mL/min/1.73 m^2^	≥60 mL/min/1.73 m^2^
Bilirubin	9 μmol/L	21 μmol/L	≤21 μmol/L
Albumin	39 g/L	38 g/L	38–48 g/L
Protein	70 g/L	73 g/L	62–80 g/L
Lactate dehydrogenase	182U/L	ND	<220U/L
Alkaline phosphatise	79 U/L	75 U/L	30–130 U/L
Gamma- glutamyl transpeptidase	12 U/L	14 U/L	≤60 U/L
Aspartate aminotransferase	18 U/L	16 U/L	5–55 U/L
Alanine aminotransferase	16 U/L	16 U/L	5–55 U/L

The palpable lymphadenopathy and splenomegaly were further investigated by a positron emission tomography (PET) scan that demonstrated increased metabolism in the spleen and mild-to-moderately increased metabolism in the bone marrow. Metabolism was also increased in lymph nodes above and below the diaphragm, many of which were not enlarged. These findings were reported as being most consistent with a well-differentiated lymphoproliferative disease process. The patient had attended an outpatient CT of the chest, abdomen and pelvis eight days prior to admission which found mild splenomegaly at 13.5 cm but no evidence of pathologic lymphadenopathy.

A bone marrow aspirate and trephine was performed to investigate for haematological malignancy or occult infection such as *Leishmania*. The bone marrow was hypercellular with moderate tri-lineage dysplasia, reported as consistent with HIV infection. There was no evidence of bone marrow involvement by a lymphoproliferative neoplasm. The patient’s symptoms improved with supportive intravenous fluid and he was discharged after ten days with an outpatient Haematology appointment and a plan to further investigate for an underlying lymphoproliferative disease, however he did not attend this appointment.

**Fig. 2:**
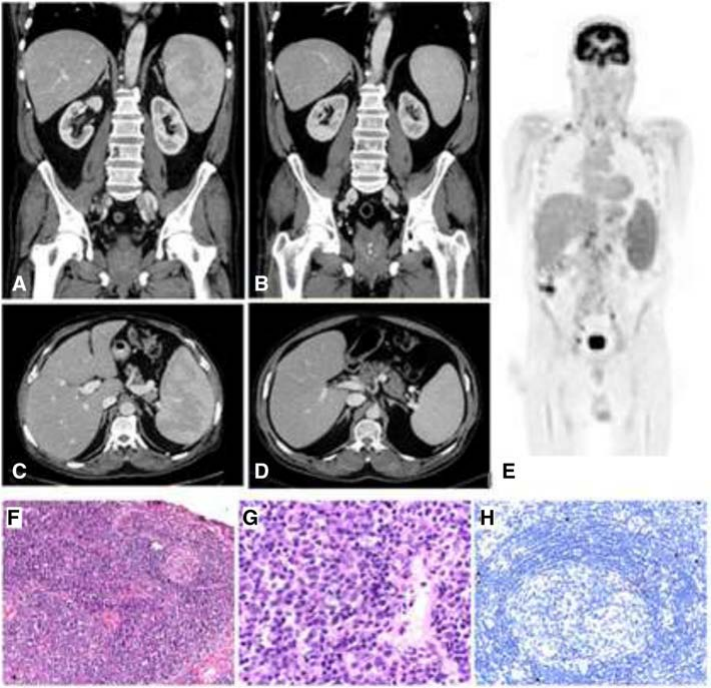
Histology of core biopsy from supraclavicular lymph node and radiological findings including a diagnostic FDG-positron emission tomography (PET) scan and comparative abdominal computed tomography before and after rituximab monotherapy. **a** PET scan performed during Admission #5 demonstrates widespread avid lymphadenopathy prior to the diagnosis of multi-centric Castleman’s disease (MCD). Images (**b**) and (**c**) show splenomegaly on computed tomography (CT) also during Admission #5. Images (**d**) and (**e**) were taken following four doses of rituximab therapy. The spleen has decreased in size from a maximum length of 15.5 cm (superioinferiorly) to 12.9 cm. **f** Low power showing regressed germinal centre and expanded interfollicular zones (H&E, x 100). **g** High power of interfollicular zone showing plasma cell proliferation (H&E, x 400). **h** Immunohistochemical staining for HHV-8 shows nuclear expression in isolated cells in the mantle zone (x 200)

Over the following 4 months, between December 2013 and March 2014, the patient had three hospital admissions each characterised by fevers >38 °C, generalised abdominal pain, urinary frequency and a CRP >100 mg/L (Fig. 1). Each of these admissions was managed as presumed urosepsis on the basis of fever, abdominal pain and urinary frequency and involved treatment with empirical intravenous antibiotics. However, numerous blood and urine cultures taken on each occasion failed to isolate a pathogen. Admission #2 was seven days long. During this time the patient had two sets of blood cultures and one urine culture which showed no growth prior to treatment with IV ceftriaxone and gentamicin. A CT of the renal tract showed left perinephric stranding with no features of obstruction, reported as possibly consistent with a recent calculus. Admission #3 (twelve days duration) included four sets of negative blood cultures and two negative urine cultures. All were taken prior to IV ciprofloxacin therapy. During this admission the patient had a systolic blood pressure of 78 mmHg requiring IV fluids in an ICU. During admission #4 (eleven days duration) the patient was hypotensive on admission (95/40 mmHg), but responded to IV fluids. He had four negative blood cultures and two negative urine cultures prior to treatment with IV ceftriaxone and gentamicin. He was commenced on tamsulosin, prophylactic sulfamethoxazole-trimethoprim 400/80 mg daily and referred for outpatient cystoscopy for further investigation of recurrent urosepsis. In January 2015 the patient re-presented to the Emergency Department with urinary frequency and loin pain (Fig. 1, Admission #5). He was febrile (38.1 °C) with flank tenderness. He was again admitted with presumptive urosepsis and commenced on IV ceftriaxone and gentamicin. A CT of the renal tract was performed and found a left hydroureter and abdominal lymphadenopathy. The patient remained febrile, however antibiotics were ceased on Day 3 after urine and blood cultures found no growth and clinical suspicion for an underlying neoplastic process was high. A PET scan was performed, this time demonstrating multiple active lymph nodes above and below diaphragm considered suggestive of a slowly progressing well-differentiated lymphoma (Fig. 2e). A CT chest, abdomen and pelvis showed splenomegaly (Fig. 2a, c), right supraclavicular adenopathy, peripancreatic adenopathy and mild retrocaval adenopathy. The patient underwent an ultrasound guided core biopsy of the right supraclavicular node. The patient defervesced and he was discharged on Day 12 with plans for outpatient follow-up.

The histopathology of his excised lymph node found preserved architecture with expanded interfollicular areas including numerous mature, polyclonal plasma cells. Immunohistochemistry for HHV-8 showed occasional positive staining cells in the mantle zone (Fig. 2f–h). These findings were reported as diagnostic of the plasma cell variant of Castleman’s disease. A plasma IL-6 level was elevated at 29.6 pg/mL (reference range <6.0 pg/mL) and HHV-8 DNA was detected in the serum in a qualitative assay as quantitation was unavailable.

The patient presented to the Emergency Department again 4 weeks later, prior to his outpatient follow-up, with a three-day history of dysuria, frequency and flank pain (Fig. 1, Admission #6). The patient was febrile to 38.6 °C, hypotensive (98/66 mmHg) and tachypnoeic, meeting the criteria for SIRS [14]. His abdomen was distended with palpable hepatosplenomegaly. Laboratory values are shown in Table 1. A CT of the renal tract was performed and reported no identifiable calculus however there was bilateral peri-nephric and peri-ureteric fat stranding with mild dilatation of both ureters. IV vancomycin and gentamicin were commenced to cover for urosepsis. The patient remained hypotensive and unstable despite fluid resuscitation, necessitating transfer to an ICU.

The patient was commenced on single strength noradrenaline (SSNAdr) initially requiring 32 mL/hr to maintain a mean arterial pressure (MAP) of >65 mmHg. He received 1 L of 4 % albumin. This reduced his SSNAdr requirements to 12 mL/hr and increased his urine output from 0 to 15 mL/hr to 60 mL/hr.

A renal DTPA-scan was performed and found normal renal perfusion but minimal renal function, a result likely confounded by use of noradrenaline. An intravenous pyelogram again made mention of bilateral perinephric stranding and the absence of hydronephrosis. An abdominal ultrasound was performed to exclude a biliary cause of sepsis. No cause for the patient’s clinical deterioration could be identified. A total of three sets of blood cultures and two urine cultures were obtained. No growth was reported in any of these specimens. While there was initial uncertainty surrounding attributing the patients fevers, flank pain and hypotension to the recently diagnosed haematological disorder, ultimately the cause for the patient’s symptoms was considered to be progressive MCD as previously described [15]. His SSNAdr infusion was ceased on Day 3 by which time the patient had been afebrile for 48 h. The patient was transferred to the ward on Day 6 of admission and was discharged on Day 12 with outpatient follow-up, with an application for use of rituximab therapy submitted.

The patient was subsequently treated with rituximab monotherapy infusions at 375 mg/m^2^ for four doses weekly which was well tolerated. A progress CT found significant reduction in the size and number of the prominent lymph nodes and a reduction in splenomegaly (Fig. 2b,d). A serum IL-6 level taken 3 weeks after the final rituximab dose had decreased from a pre-treatment level of 29.6 pg/mL to 0.9 pg/mL (reference range <6.0 pg/mL). The patient remains clinically stable and regularly attends follow-up clinics. There have been no further admissions characterised by fever and/or hypotension.

## Conclusions

Patients with MCD present with heterogenous symptoms of a systemic inflammatory response with the most common features being: malaise, fever, night sweats, weight loss, generalised lymphadenopathy, splenomegaly, oedema and cytopenias [2, 3, 16]. It is now acknowledged that cytokine dysregulation, largely driven by IL-6, is responsible for amplifying this systemic response.

Whereas MCD is known to have a higher incidence in HIV-infected individuals and generally occurs in those with a CD4 T-cell count <0.35 x 10^9^ cells/L, unlike Kaposi’s sarcoma there appears to be no correlation between relapse of MCD and CD4 T-cell count or the use of ART [17]. In a systematic review of HIV-associated MCD totalling 68 patients, the median age was 40 years, 90 % of cases occurred in males and 82 % were in Caucasians [3]. The median time between HIV diagnosis and MCD diagnosis for patients in the combined ART era was 33 months and the median CD4 count at diagnosis was 0.156 x 10^9^ cells/L [3]. Of 48 patients diagnosed in the combined ART era, 68 % were already on HIV treatment at the time of diagnosis [3]. Indeed, some data suggests an increase in the incidence of MCD in the post-ART era, in contrast to Kaposi’s sarcoma [17].

There are no current diagnostic criteria for MCD disease activity however the presence of fever and a raised CRP appear important. Furthermore, there are no evidence-based criteria for diagnosing HIV-associated MCD. Previous analyses found the most common presenting symptom in HIV-associated MCD was fever (100 %) with the majority having lymphadenopathy (95 %), splenomegaly (86 %) and hepatomegaly (62 %) [3]. The most consistently reported laboratory finding is of a significantly elevated CRP [5], which was evident during each of our patients admissions (Fig. 1). Other reported lab findings include polyclonal hypergammaglobulinemia, hypoalbuminemia, anaemia and pancytopenia [5]. The French Agence Nationale de Recherche sur le SIDA 117 CastlemanB group have proposed criteria for the diagnosis of HIV-associated MCD, which require fever, CRP >20 mg/L and at least three of the following clinical features: peripheral lymphadenopathy, splenomegaly, oedema, pleural effusion, ascites, cough, nasal obstruction, xerostomia, rash, central neurologic symptoms, jaundice and autoimmune haemolytic anaemia. Interestingly, our patient does not satisfy the criteria having only had lymphadenopathy and splenomegaly. However, the features on histopathology were diagnostic for MCD. Additionally, while our patient displayed a drop in haemoglobin below the lower limit of normal during each admission (Fig. 1), there were no other features of haemolysis with bilirubin, lactate dehydrogenase (Table 1), haptoglobin and direct antibody test all within normal limits. This indicates that there are likely additional mechanisms for anaemia in MCD. Plasma HHV-8 DNA and plasma IL-6 levels may be useful additional screening tools for MCD, however these have been yet to be validated in clinical trials [6].

This case highlights the difficulty in making a diagnosis of MCD largely due to heterogeneous symptoms and signs that may wax and wane and/or mimic other conditions such as sepsis. Certainly due to such difficulties it is likely that there is often a delay between onset of symptoms and diagnosis, and that the condition is under diagnosed [18]. Our patient is typical for HIV-associated MCD in being male, and having a relatively low CD4 T-cell count, however he is older than the median age of 44 years. Additionally, his HIV diagnosis was longer than the median of 3 years. There are, however, similarities between our patient and a previous report by Lederer et al. [15], who describe a 55 year old man with chronic HIV infection for 3 years virologically controlled on ART with a CD4 T-cell count of 0.107 x 10^9^ cells/L. The previous case also involved multiple presentations to hospital with fever, (as well as weakness and diarrhoea), elevated CRP to 228 mg/L and hypotension to 95/54 mmHg. However, unlike this case the former did not require ICU admission or ionotropic support.

This is the first report of hypotension and vasodilatory shock requiring ICU admission and ionotropic support in the setting of HIV-associated MCD. It is most likely that the hypotension associated with both this case and that of Lederer et al. [15] was related to active HIV-associated MCD. Additionally it is the first report of MCD mimicking urosepsis. The patient’s major complaint of urinary frequency at each admission contributed to an erroneous provisional diagnosis of urinary tract infection. It is unclear how our patient’s subjective urinary frequency related to the radiological findings of perinephric stranding and if this could be related to IL-6-mediated inflammation around the urinary tract. There is little published data on the relationship between IL-6 and the urinary tract and it will be interesting to discover if urinary frequency is a feature of future cases of MCD.

While a number of therapeutics are used to treat MCD, none are curative and the goal is to control the disease and symptoms. Treatment options include the chemotherapeutic agents cyclophosphamide, doxorubicin, vincristine, prednisone (CHOP), antiviral agents, corticosteroids and biologics including the anti-CD20 monoclonal antibody rituximab, either as monotherapy or in combination with other agents.

There is no reliable randomised clinical trial data to guide the treatment of MCD. A recent study from the USA demonstrates the most common treatments for MCD were prednisone monotherapy (33 %) and sole rituximab therapy (19 % [2]). Cohort studies of rituximab at 375 mg/m^2^ weekly for 4 weeks has been shown to be safe and effective in HIV-associated MCD with 20/21 having clinical remission, haematological and serological normalisation and 70 % having a radiological response [19]. In the latter cohort study the 2-year survival was 95 % and disease free survival was 79 %. Another study found a 92 % sustained remission rate [20].

Monoclonal antibodies targeting IL-6 receptor (IL-6R; tocilizumab) or free IL-6 (siltuximab) have been trialled with the aim of interrupting the inflammatory signals from both host and virally produced IL-6. Early studies found administration of tocilizumab resolved fevers and fatigue and improved inflammatory markers including CRP [21]. In a study of 28 HIV-uninfected patients tocilizumab was found to decrease lymphadenopathy and improve inflammatory markers [22], however only two patients were HHV-8+. Blockade of IL-6 has also shown some promise in achieving clinical and laboratory normalisation [23]. In a study of 37 patients with MCD 32/37 (87 %) obtained a clinical benefit response in a least one measure, however only 1/37 achieved a complete response and 11/37 had a partial response [24]. The use of tocilizumab in HIV-associated MCD is currently limited. Interestingly in a report of two cases, tocilizumab was effective at inducing short-term resolution of symptoms and elevated CRP, but patients exhibited relapse of MCD after 15 and 22 weeks prompting second line treatment with rituximab, which induced clinical remission in both cases for 1–4 years [25]. There appears to be an emerging consensus that rituximab with or without etoposide is appropriate first-line therapy for HIV associated MCD [6]. The mechanism of rituximab’s action here is unclear as the majority of HHV-8+ plasmablasts lack surface CD20 (discussed in [18]).

Despite the rarity of HIV-associated MCD and the lack of consensus guidelines on diagnosis and treatment, some progress has been made in the management of this disease. In a study of 86 cases between 1985 and 2006 the median survival was 12 months [3]. However more recent data from the USA, reported 92 % of subjects alive at 2 years [2].

Castleman’s disease exists at a fascinating intersection between tumour biology, viral infection and immunodeficiency. This study builds on previous reports to illustrate the importance of considering the diagnosis of MCD in patients with chronic HIV infection presenting in multiple hospital admissions for sepsis-like episodes associated with fever and hypotension secondary to vasodilatory shock, especially if no infective agent is identified or if lymphadenopathy is clinically or radiologically evident. Untreated MCD can lead to significant morbidity and mortality, however identification allows for treatment with newer agents such as rituximab, which is generally well tolerated and can improve clinical outcome.

### Consent

Written informed consent was obtained from the patient for publication of this case report and any accompanying images. A copy of the written consent is available for review by the Editor of this journal.

## Abbreviations

ART: antiretroviral therapy
BD: twice daily
CD: cluster of differentiation
CHOP: cyclophosphamide doxorubicin vincristine prednisone
CMV: cytomegalovirus
CRP: C-reactive protein
CT: computer tomography
DNA: deoxyribose nucleic acid
GORD: gastro-oesophageal reflux disease
HBV: hepatitis B virus
HHV-8: human herpesvirus-8
HIV: human immunodeficiency virus
ICU: intensive care unit
IL: interleukin
KICS: Kaposi sarcoma herpesvirus inflammatory cytokine syndrome
MAP: mean arterial pressure
MCD: multicentric Castleman’s disease
PET: positron emission tomography
RNA: ribonucleic acid
SIRS: systemic inflammatory response syndrome
SSNAdr: single strength noradrenaline
USA: United States of America
